# 140-year history of pharyngoesophageal reconstruction

**DOI:** 10.1017/S0022215124000902

**Published:** 2024-10

**Authors:** Oleksandr Butskiy, Brent A Chang, Donald W Anderson, Norbert Banyi, Eitan Prisman

**Affiliations:** 1 Division of Otolaryngology – Head and Neck Surgery, Vancouver General Hospital, Vancouver, British Columbia; 2 University of British Columbia, Vancouver, British Columbia, Canada; 3 Department of Otolaryngology – Head & Neck Surgery, Mayo Clinic Arizona, Phoenix, Arizona; 4 Faculty of Medicine, University of British Columbia, Vancouver, British Columbia, Canada

**Keywords:** head and neck cancer, surgery, history of medicine, pharynx, esophagus, radiotherapy

## Abstract

**Objective:**

For over a century, circumferential pharyngoesophageal junction reconstruction posed significant surgical challenges. This review aims to provide a narrative history of pharyngoesophageal junction reconstruction from early surgical innovations to the advent of modern free-flap procedures.

**Methods:**

The review encompasses three segments: (1) local and/or locoregional flaps, (2) visceral transposition flaps, and (3) free-tissue transfer, focusing on the interplay between pharyngoesophageal junction reconstruction and prevalent surgical trends.

**Results:**

Before 1960, Mikulicz-Radecki's flaps and the Wookey technique prevailed for circumferential pharyngoesophageal junction reconstruction. Gastric pull-up and colonic interposition were favoured visceral techniques in the 1960s–1990s. Concurrently, deltopectoral and pectoralis major flaps were the preferred cutaneous methods. Free flaps (radial forearm, anterolateral thigh) revolutionised reconstructions in the late 1980s, yet gastric pull-up and free jejunal transfer remain in selective use.

**Conclusions:**

Numerous pharyngoesophageal junction reconstructive methods have been trialled in the last century. Despite significant advancements in free-flap reconstruction, some older methods are still in use for challenging clinical situations.

## Introduction

**Figure 1.**
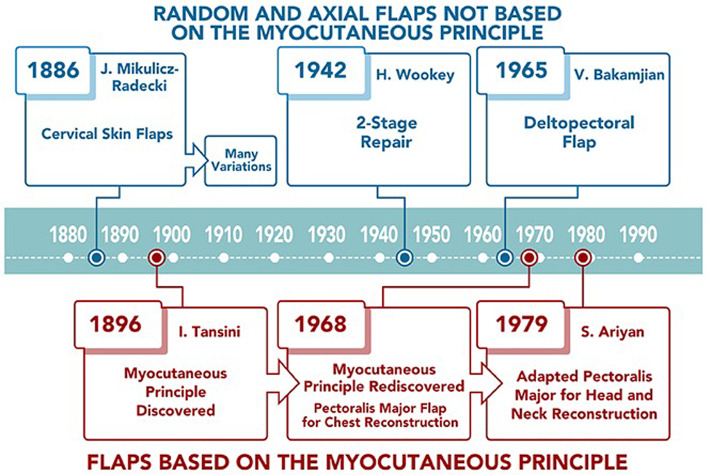
Historical overview of local and locoregional flap pharyngoesophageal reconstruction.

Circumferential resection of the pharyngoesophageal junction is reserved for advanced malignancies of the hypopharynx, cervical oesophagus, larynx or thyroid, and occasionally for severe caustic injuries of the upper aerodigestive tract.^1–3^ Reconstruction of the resulting defect (Figure 1) has remained a major surgical challenge for over a century.^4^ Given the lack of high-quality evidence to support one pharyngoesophageal reconstruction technique over the other, a historical perspective of pharyngoesophageal reconstruction is essential to understand the modern techniques and the problems they were designed to address. The last review of the history of pharyngoesophageal reconstruction is 40 years old and predates the popularisation of microvascular reconstruction.^4^ The purpose of the present review is to provide an updated historical perspective of circumferential pharyngoesophageal reconstruction.

The various reconstructive options trialled over the years are difficult to categorise. For the purposes of this review, the techniques are grouped into three categories: (1) local and locoregional cutaneous and myocutaneous flaps, (2) pedicled visceral flaps, and (3) free-tissue transfer.

## Local and locoregional flaps (Figure 1)

The first experimental work on the resection of “the upper gullet” was performed in the surgical school of Theodor Billroth in Vienna in the late nineteenth century. In 1871, Professor Billroth resected the upper oesophagus in a series of dogs and closed the resulting defect by pulling the distal oesophagus up and creating a primary anastomosis.^5^ Billroth's pupil, Vincenz Czerny, performed the first resection of a tumour posterior to the larynx in 1877. Czerny was unable to close the resulting defect, which soon proved impossible for anything other than small tumors.^6,7^ Czerny's patient survived for one year.

Ten years following the first cervical oesophageal resection, another one of Billroth's pupils, Jan Mikulicz-Radecki, re-established alimentary continuity by surgically folding cervical skin flaps.^8^ In the first decade of the twentieth century, Mikulicz's method was adopted and modified by a variety of European surgeons.^9–12^ These modifications required three to four surgeries, which resulted in unacceptably high morbidity and mortality in the pre-antibiotic era.

The field of radiation oncology was advancing concurrently and only four years after the 1898 discovery of polonium and radium by Marie and Pierre Curie, pharyngeal carcinoma was successfully treated with radiation in Vienna.^13^ More patients were being treated with radiation and the irradiated cervical skin flaps were less reliable for reconstruction. Radiation became the primary treatment and palliation of cancers at the pharyngoesophageal junction at the time.^14,15^

**Figure 2.**
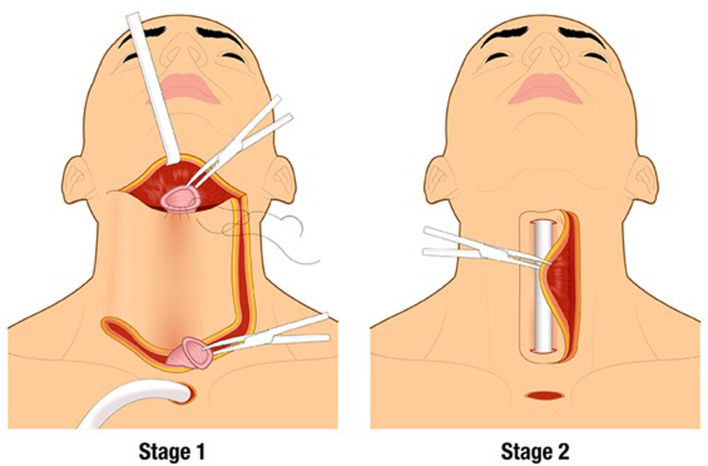
Wookey technique for two stage closure of the pharyngoesophageal defect with cervical skin flaps.

Surgical enthusiasm was renewed during World War II.^16^ In 1942, Harold Wookey of the University of Toronto developed a two stage cervical oesophageal reconstruction: in the first operation, a cervical pharyngotomy was created, and in the second operation, the pharyngotomy was closed by folding laterally based cutaneous flaps (Figure 2).^17^ This technique was modified several times and it remained the standard of care until the 1960s, but still often used irradiated skin. The Wookey technique is used today in patients who have exhausted other reconstructive options.^18^

**Figure 3.**
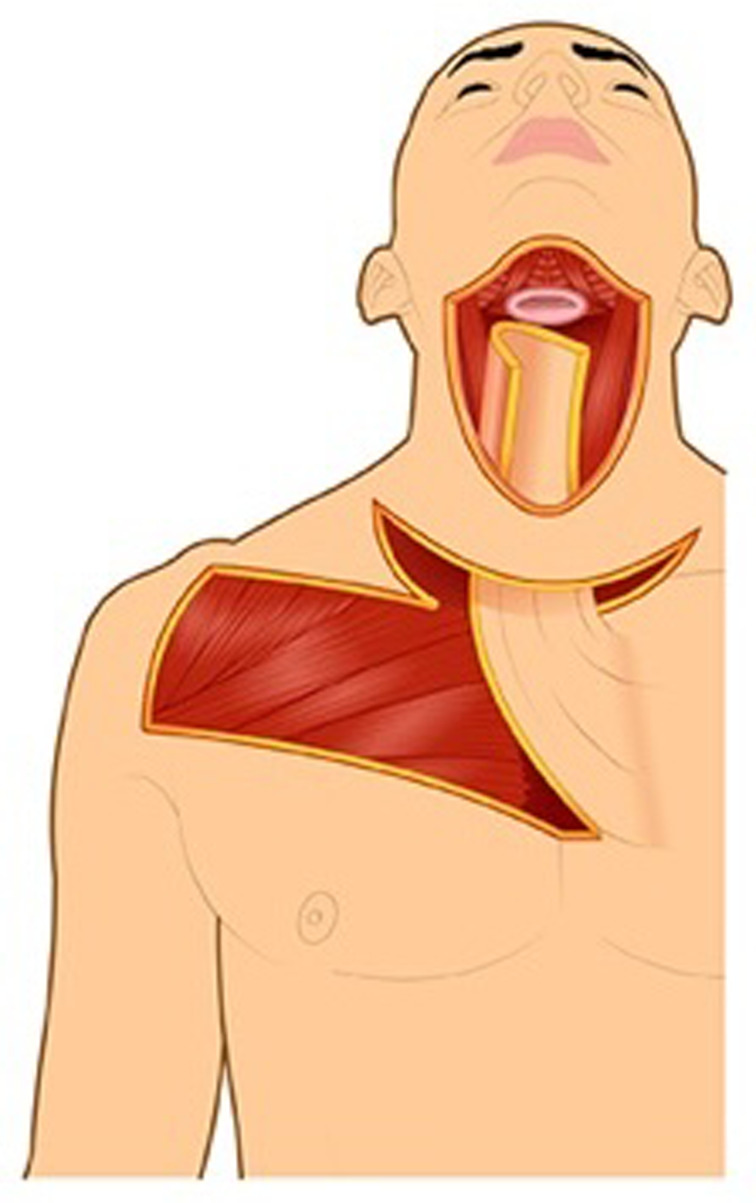
Deltopectoral flap, stage 1.

An obvious donor site for the pharyngoesophageal reconstruction outside the irradiated field is the chest. Thoracic skin flaps used for pharyngoesophageal reconstruction can be classified into random or axial flaps. Random flaps were used extensively for pharyngoesophageal reconstruction in the 1950s.^19–21^ Unlike random flaps, axial flaps rely on knowledge of the cutaneous blood supply, which was described in 1889 by Manchot.^22^ The first axial flaps were demonstrated by Davis in 1919.^23^ It was not until 1965 that Vahram Bakamjian introduced the first clinically useful axial flap: the deltopectoral flap (Figure 3).^24^

The deltopectoral flap was a conceptual breakthrough in flap design, increasing the reliability of the donor skin as compared to random flaps. Bakamjian specifically designed the deltopectoral flap to address the problem of pharyngoesophageal reconstruction.^24^ Unfortunately, like the Wookey flap, the deltopectoral flap required a two-stage reconstruction. In 1969, Harrison found that with multi-step pharyngoesophageal reconstruction methods, local recurrence was seen between stages in at least 50 per cent of his patients.^25^

Prior to the 1970s, there were multiple attempts to solve the problem of multiple reconstructive stages with the use of laryngotracheal autografts,^26,27^ free-skin grafting over stents,^28^ and the use of plastic tubes.^29^ One intriguing idea was the use of free, full-thickness tubular grafts of penile skin.^30^ These were complex procedures complicated by oral microbial flora contaminating the surgical fields, frequently resulting in wound breakdown, fistula formation or carotid blowout.^4^

The conceptual breakthrough that allowed successful single-stage reconstruction of pharyngoesophageal defects with pedicled cutaneous flaps came in the late 1960s using the rediscovered myocutaneous flap principle. Remarkably, the myocutaneous flap principle was pioneered at the end of the nineteenth century by Iginio Tansini,^31^ an Italian contemporary of Professor Cherny. Because myocutaneous flaps do not require a separate skin pedicle for survival, the cutaneous portion of the myocutaneous flap can be tubed in a single operation (Figure 5).

**Figure 4.**
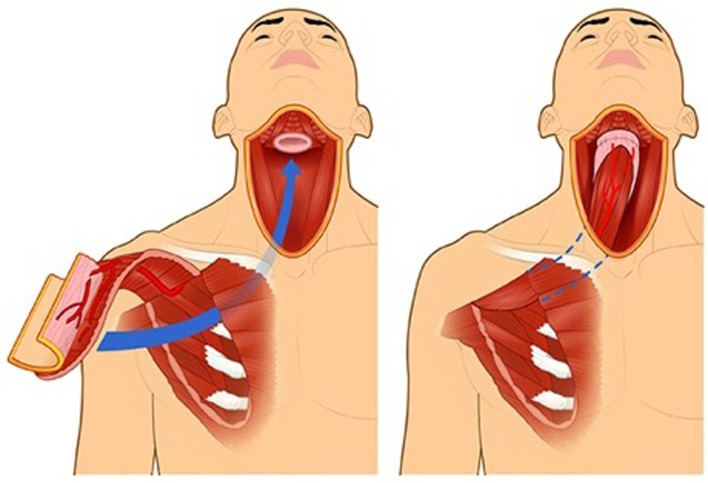
Pectoralis major flap used for pharyngoesophageal reconstruction.

The first myocutaneous flap adopted for head and neck reconstruction was the pectoralis major flap (Figure 4). Pioneered by an Australian thoracic surgeon for chest reconstruction in 1968,^32^ its utility for head and neck reconstruction was not recognised until 1979 by Ariyan.^33^ By the early 1980s, some considered the myocutaneous pectoralis flap to be the “ideal form of pharyngo-oesophageal reconstruction.”^4^ It was later criticised for bulkiness and high incidence of fistula formation.^34,35^ A recent solution to minimise bulk is the partial pectoralis major flap tubing, incorporating prevertebral fascia into circumferential pharyngeal reconstruction.^36^

In the past 25 years, the use of local and locoregional cutaneous flaps for pharyngoesophageal reconstruction has been largely superseded by the use of free flaps. It is important to note a recent resurgence in regional pedicled cutaneous flaps for head and neck reconstruction due to their relative simplicity and ease of harvest. A reconstructive option that has received particular attention in recent literature is the supraclavicular flap.^37,38^

## Visceral transposition flaps (Figure 5)

A guiding principle of reconstructive surgery is to replace like with like.^39^ Thus, the extensive history of reconstructing the pharyngoesophageal junction with the digestive tract is expected.

**Figure 5.**
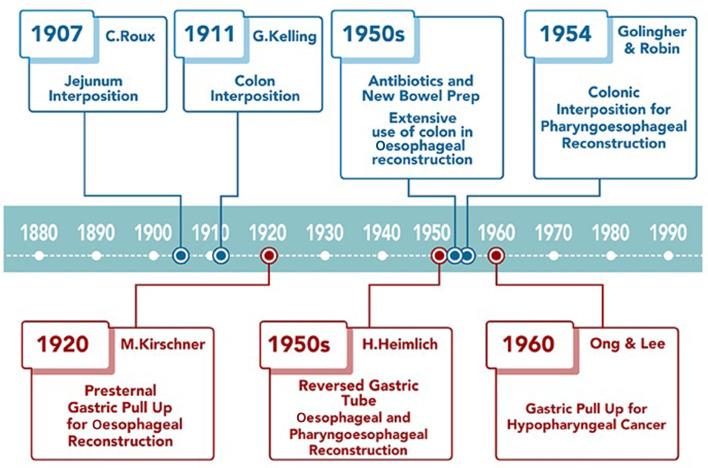
Historical overview of visceral transposition flaps for pharyngoesophageal reconstruction.

Until the development of free flaps in the 1980s, the only means of using viscera for pharyngoesophageal reconstruction was to transpose it while tethered by its vascular pedicle. In the early twentieth century, abdominal viscera were transposed into the neck by tunnelling either anterior or posterior to the sternum. This often resulted in limited deglutition, particularly when tunnelled anterior to the sternum, where patients would have to physically “milk” the bolus towards the stomach. A significant advance was contributed by Ivor Lewis in 1946 who developed the technique of right-sided thoracotomy for esophagectomy.^40^ This technique facilitates transposing the viscera through the oesophageal bed to the neck following an oesophagectomy.^41^ Three abdominal organs have been used in the pharyngoesophageal reconstruction: jejunum, colon and stomach.

### Jejunum

The jejunum's isoperistaltic activity made it an attractive option for oesophageal reconstruction. The first reports of oesophageal reconstruction with a pedicled jejunum were published independently by Roux^42^ and Herzen^43^ in 1907. These were multistage operations with two main challenges preventing its use in mainstream practice: (1) the vascular pedicles and vascular arcades were distant from the bowel edge, making survival of the transposed jejunum tenuous; and (2) redundant loops of bowel in the chest led to frequent obstruction.^4^

### Colon

**Figure 6.**
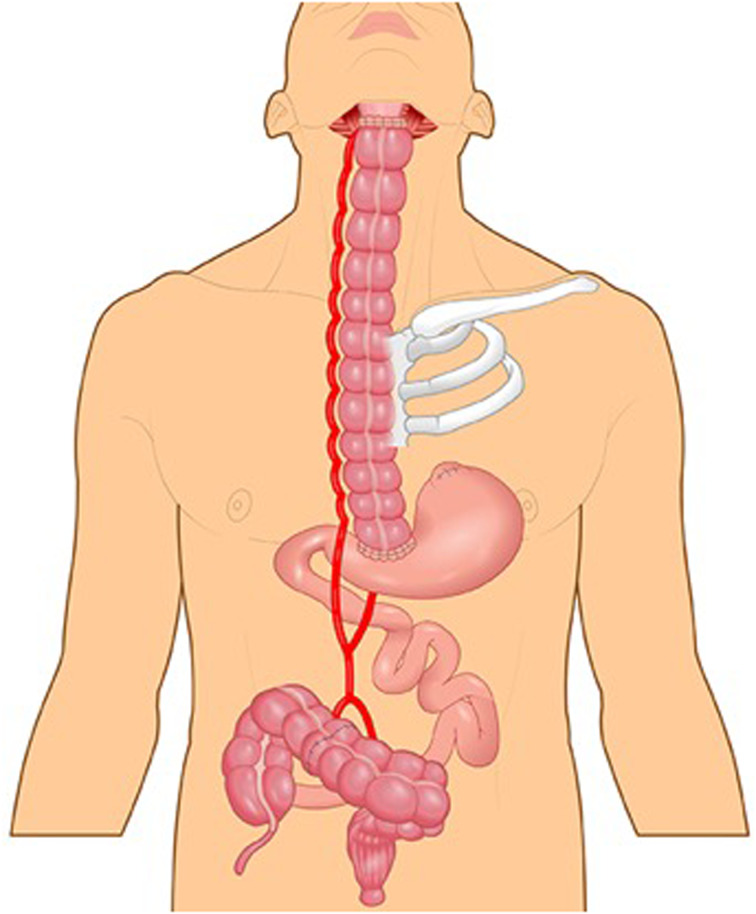
Left colon supplied by the middle colic artery and used for pharyngoesophageal reconstruction.

The use of pedicled colon for pharyngoesophageal reconstruction proved to be more reliable than jejunum and is currently used in select circumstances. In 1911, Georg Kelling performed the first reconstruction of the oesophagus with the pedicled transverse colon.^44^ In 1954, Goligher and Robin used antibiotics to conduct the first successful pharyngoesophageal reconstruction with the left colon supplied by the middle colic artery (Figure 6).^45^ The advantages of this technique were the reliable blood supply, resistance of colonic mucosa to gastric secretions and the resistance to stenosis. However, the number of anastomotic connections and the extent of surgery increased morbidity. Hence, this procedure was used as a primary option for pharyngoesophageal junction reconstruction only in a few centres briefly in the mid to late 1950s.

### Stomach

**Figure 7.**
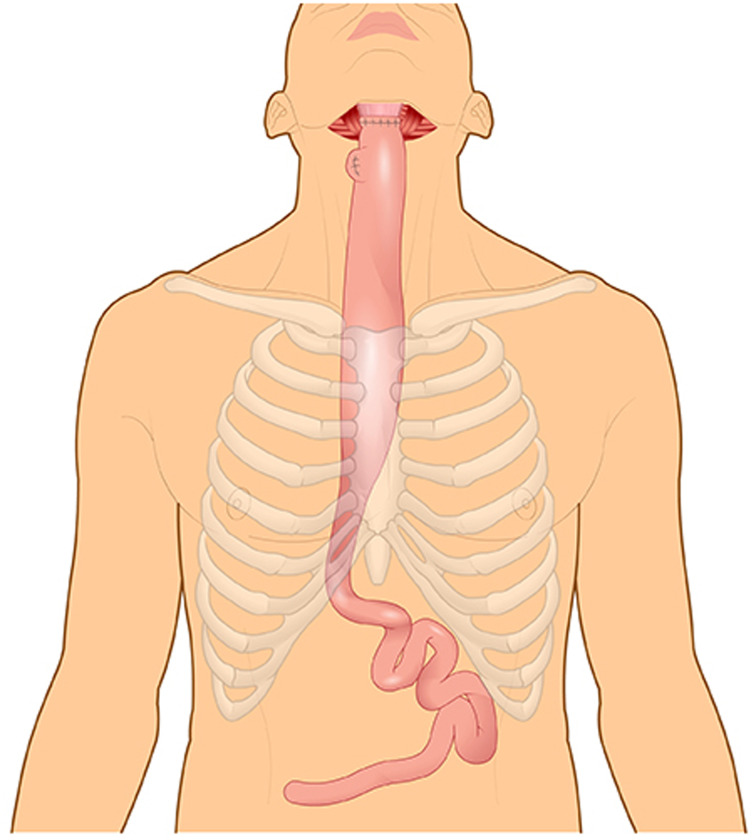
Gastric pull-up.

Martin Kirschner was the first surgeon to replace the thoracic oesophagus with mobilised stomach. In 1920, Kirschner successfully treated a patient with a lye stricture by bypassing the stricture with the mobilised stomach tunnelled subcutaneously in front of the sternum.^46^ A few years later, Kirschner's operation was modified by tunnelling the stomach through the oesophageal bed.^47^ This operation became known as the gastric pull-up, which remains the standard of care for thoracic oesophageal cancer (Figure 7).^48^

**Figure 8.**
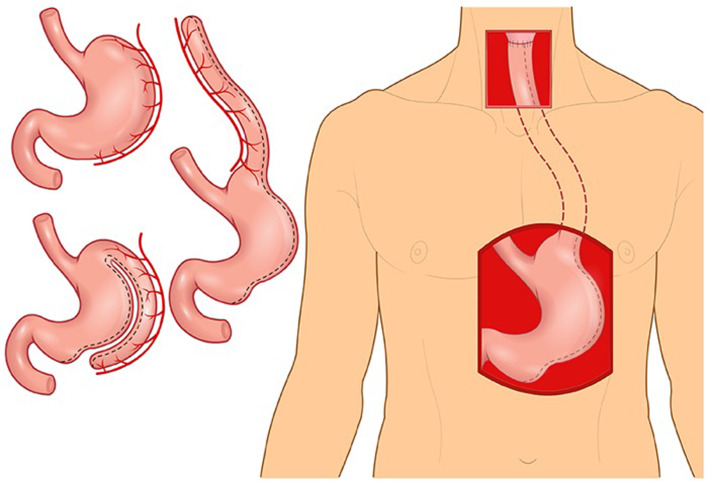
Reversed gastric tube used for pharyngoesophageal reconstruction.

In the 1950s, surgeons feared that gastric pull-up for pharyngoesophageal junction reconstruction would not allow tensionless anastomosis.^45^ To overcome this potential problem some surgeons advocated for the reversed gastric tube,^49^ which had been investigated in dogs by Beck and Jianu at the turn of the twentieth century.^50,51^ Fifty years after the original experiments, Henry Heimlich was the first to apply the reversed gastric tube for pharyngoesophageal junction reconstruction, but it subsequently was seldom used due to high morbidity (Figure 8).^4,49^

GB Ong and TC Lee from Hong Kong challenged the notion that gastric pull-up would not reach the pharynx and performed it in 1959 following pharyngo-laryngo-oesophagectomy for three patients, demonstrating adequate length to reach the pharynx.^52^ They reconstructed the pharyngoesophageal junction defect in one operation and returned their patients to a normal diet as early as 10 days after surgery.^52^ No other operation at that time was capable of returning patients to eating as quickly.^53^

The success of GB Ong and TC Lee and the absence of alternative reconstructive methods explained the rapid rise in popularity of the gastric pull-up for pharyngo-oesophageal reconstruction in the 1960s. Surprisingly, the gastric pull-up has undergone minimal modification since. In the mid 1960s, the transhiatal oesophagectomy replaced routine thoracotomy for oesophagectomy.^54^ More recently, several authors have tried thoracoscopic oesophagectomy^3, 55, 56^ and laparoscopic approaches for stomach mobilisation.^57–60^. It is unclear if these methods result in improved patient outcomes.

In the late 1970s and early 1980s, alternatives to the gastric pull-up were developed and the enthusiasm for gastric pull-up waned due to reports of high morbidity and mortality. Some reports quoted mortality rates of close to 50 per cent.^61,62^ In 1986, writing about the gastric pull-up, Harrison stated that “with many other alternatives there could be no justification for carrying out what appears to be, in some reports, surgical euthanasia.”^63^

A more recent review showed gastric pull-up post-operative mortality has decreased and is approaching that of other pharyngoesophageal reconstruction techniques in high volume centres.^64^ Gastric pull-up continues to be used, mostly in cases where the surgical defects extend below the thoracic inlet. Free-flap reconstruction, which was developed and popularised in the late 1980s and early 1990s, has now replaced both the gastric pull-up and the pedicled myocutaneous flaps as the method of choice for pharyngoesophageal reconstruction.

## Free-tissue transfer (Figure 9)

Development of free-tissue transfer was in part spurred by the challenges posed by pharyngoesophageal reconstruction. Prior to the use of myocutaneous flaps, no method offered a reliable tubed cutaneous conduit in one operation. A potential solution was to auto-transplant free, non-vascularised abdominal viscera, such as jejunum, to the neck. This procedure was experimentally investigated in a series of mongrel dogs by Bernard Seidenberg from New York in the late 1950s.^14^ Steinberg's group also performed the first clinical free-jejunal reconstruction of the hypopharynx in 1957.^14^ This was soon followed by free transfer of the gastric antrum in 1961,^65^ and of the sigmoid colon in 1964.^66^ Out of the visceral free flaps, only the free-jejunum flap continues to be used for pharyngoesophageal reconstruction. Free-jejunal reconstruction provides relatively low anastomotic leak rate, with high volume centres reporting leak rates as low as 5.2 per cent,^67^ and allows up to 90 per cent of patients to be maintained on an oral diet.^37^ The major drawbacks of the jejunal free flap are the “wet voice” from mucous secretions by the flap and the morbidity of the donor site.^37^

- From the late nineteenth century to the 1960s, pharyngoesophageal junction reconstruction relied on local skin flaps (Mikulicz-Radecki's and Wookey's techniques), requiring multiple surgeries
- In the 1950s, colonic interposition and gastric pull-up were introduced; improvements over the past two decades have made gastric pull-up a continued choice for tumours extending into the thoracic oesophagus
- Ionising radiation was the primary treatment for pharyngoesophageal junction malignancies until the 1960s, so local skin flaps were complicated by the use of radiated tissues
- In 1965, the deltopectoral flap advanced reconstruction by enabling a two-stage procedure with better blood supply, using tissue not affected by radiation
- During the 1980s, the pectoralis major myocutaneous flap enabled single-stage reconstruction
- Free-tissue transfers, particularly tubed radial forearm and anterolateral thigh flaps, became preferable for pharyngoesophageal junction repair in the 1980s and the free jejunal flap emerged as an alternative to colonic interposition and gastric pull-up
- Despite advancements in free flap reconstruction, older methods are still in use for challenging clinical situations.

**Figure 9.**
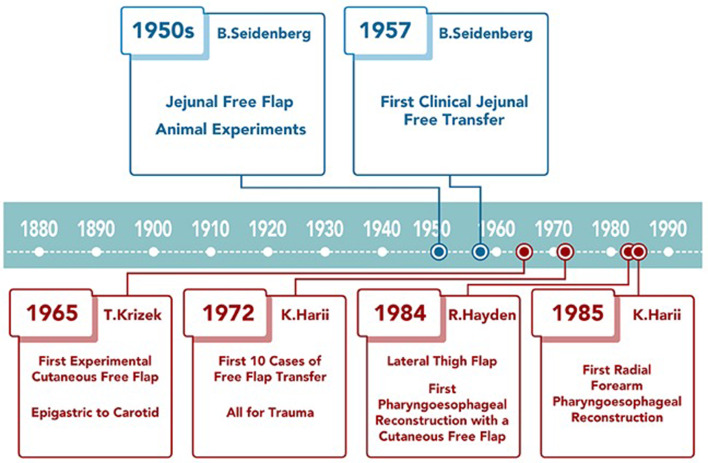
Overview of free-tissue transfer history and its application for pharyngoesophageal reconstruction.

The successes with microsurgical experiments and with free visceral transfers in the 1960s laid the foundation for the development of composite cutaneous free flaps. The first successful composite free-flap transfer was performed in a canine model by Krizek *et al*. in 1965.^68^ There is some controversy over who performed the first successful human microvascular composite tissue auto transplantation,^69^ but most credit Harii from Japan in 1972.^70^ Over the next decade, a number of free-tissue transfer options were developed.^71^ It was not until the mid to late 1980s that free-tissue transfer was widely practiced and once its reliability was shown,^72^ it became the standard of care for head and neck oncologic surgical defects.

The first reported free-tissue pharyngoesophageal reconstruction was by Hayden *et al*. in 1984, using the lateral thigh fasciocutaneous flap.^73^ The major advantages of free-tissue transfer for pharyngoesophageal reconstruction are one-stage reconstruction, relatively low morbidity to the donor site, and avoidance of complications associated with entering the abdominal cavity or mediastinum. However, the cutaneous tubes are known to be more prone to leaks and strictures than the visceral options.^74–76^ In the last 20 years, the radial forearm free flap and anterolateral thigh flap have been used most frequently for pharyngoesophageal reconstruction.^37^

The radial forearm free flap flap was developed and popularised in China in the early 1980s.^77^ The first reported case of pharyngoesophageal reconstruction with the radial forearm free flap was by Harii *et al*. in 1985.^78^ Harii's team popularised the trapezoid design of the flap folded on itself, with the proximal end wider than the distal end, creating a funnelled tube with a longitudinal suture line. Early experiences with this flap showed higher frequency of salivary leaks and fistulas compared to the jejunal free flap, which was popular at the time.^74–76^ Several modifications to reduce the frequency of anastomotic leaks included use of the Montgomery salivary bypass stent^79^ and de-epithelialisation of the vertical suture line.^80^

**Figure 10.**
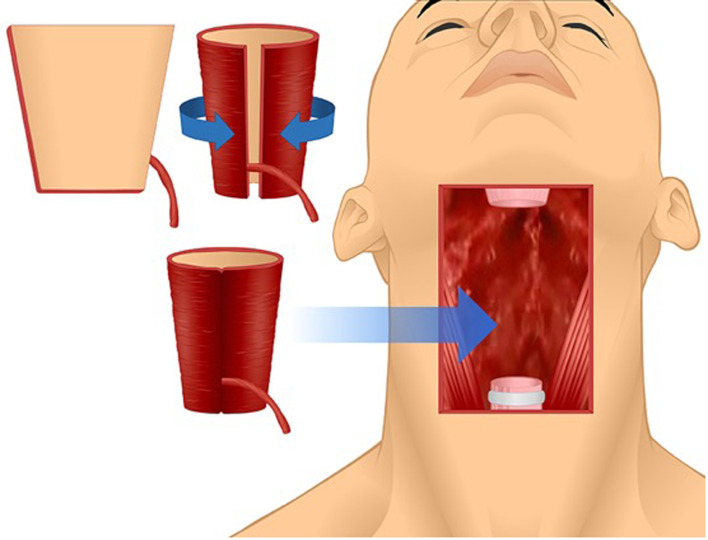
Anterolateral thigh free flap folded into a cone and used for pharyngoesophageal reconstruction.

The anterolateral thigh flap is another fasciocutaneous flap that is now routinely used for pharyngoesophageal reconstruction. The anterolateral thigh flap, described by Song *et al*. in 1984,^81^ has gained significant popularity for head and neck reconstruction (Figure 10).^37^ One of the often quoted advantages of the anterolateral thigh flap over the radial forearm free flap is that the fascial layer of the anterolateral thigh flap can wrap around the suture line and protect it from salivary leaks.^37^ In recent years, surgical centres worldwide have been trending towards favouring the anterolateral thigh flap over other forms of cutaneous free flap pharyngoesophageal reconstruction.^37^ While survival of hypopharyngeal and cervical carcinomas have improved dramatically since Billroth's first experimental surgeries in the late nineteenth century, it is still estimated to be only between 15 and 56 per cent^82^.

## Summary

**Figure 11.**
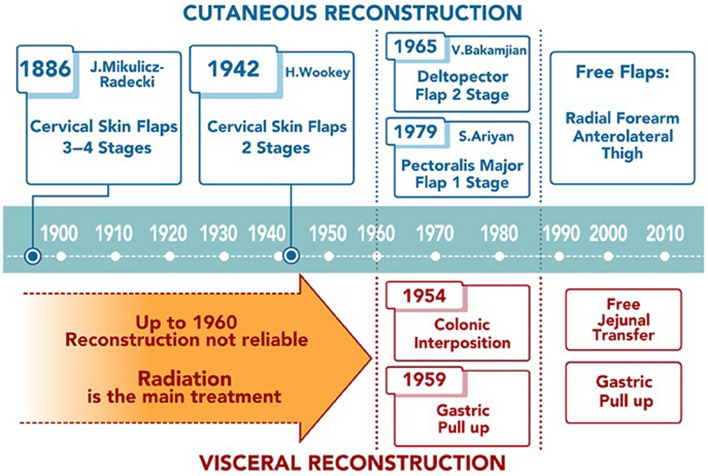
Summary of the most popular reconstructive options over the last century. Approximate periods of technological advance are separated by dashed vertical lines.

This historical review (Figure 11) provides insights into the various reconstructive techniques and their relative applications and challenges in restoring speech and swallow post-resection. From the late nineteenth century to the 1960s, pharyngoesophageal junction reconstruction relied on local skin flaps, such as Mikulicz-Radecki's and Wookey's techniques, which required multiple surgeries. Because ionising radiation was the primary treatment for pharyngoesophageal junction malignancies until the 1960s, local skin flaps were complicated by the use of radiated tissues. In the 1950s, colonic interposition and gastric pull-up were introduced. Despite early high rates of complication, improvements over the past two decades have made gastric pull-up a continued choice for tumours extending into the thoracic oesophagus. In 1965, the deltopectoral flap advanced reconstruction by enabling a two-stage procedure with better blood supply, using tissue not affected by radiation. During the 1980s, the pectoralis major myocutaneous flap enabled single-stage reconstruction. Free-tissue transfers, particularly tubed radial forearm and anterolateral thigh flaps, became preferable for pharyngoesophageal junction repair in the 1980s and the free jejunal flap emerged as an alternative to colonic interposition and gastric pull-up. Despite advancements in free-flap reconstruction, older methods are still in use for challenging clinical situations.
